# Supplementary material for: Splice-Junction-Based Mapping of Alternative Isoforms in the Human Proteome
Source: Cell Rep. Author manuscript; Available in PMC 2020 Jan 15. (PMC6961840; doi:10.1016/j.celrep.2019.11.026)
Supplement: 2 [file NIHMS1546469-supplement-2.zip › DF1/PXD009737/TestisTrypsin/TestisTrypsin_57_ABCA2_GHLPATALR.pdf]

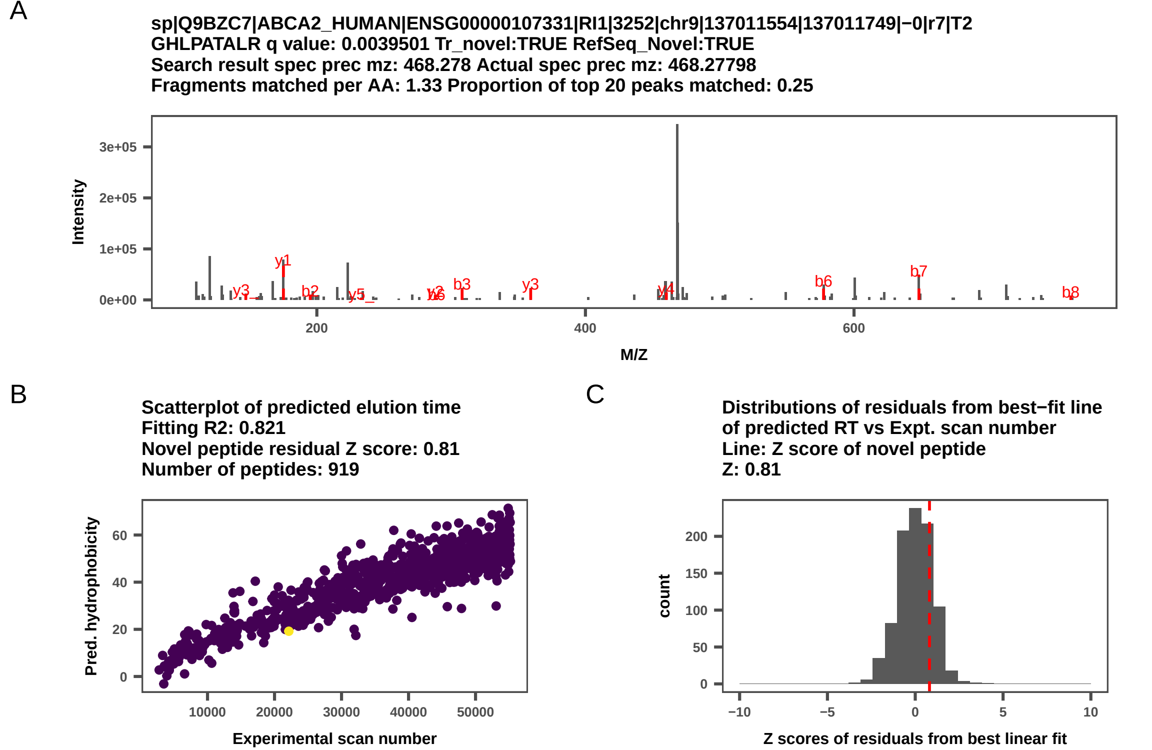
A
sp|Q9BZC7|ABCA2_HUMAN|ENSG00000107331|RI1|3252|chr9|137011554|137011749|−0|r7|T2
GHLPATALR q value: 0.0039501 Tr_novel:TRUE RefSeq_Novel:TRUE
Search result spec prec mz: 468.278 Actual spec prec mz: 468.27798
Fragments matched per AA: 1.33 Proportion of top 20 peaks matched: 0.25
Intensity
3e+05
2e+05
1e+05
0e+00
y1
y3_
b2
y5_
b3
y3
y4
b6
b7
b8
200
400
600
M/Z
B
Scatterplot of predicted elution time
Fitting R2: 0.821
Novel peptide residual Z score: 0.81
Number of peptides: 919
Pred. hydrophobicity
60
40
20
0
10000
20000
30000
40000
50000
Experimental scan number
C
Distributions of residuals from best−fit line
of predicted RT vs Expt. scan number
Line: Z score of novel peptide
Z: 0.81
count
200
150
100
50
0
−10
−5
0
5
10
Z scores of residuals from best linear fit
